# Regulation of CAR and PXR Expression in Health and Disease

**DOI:** 10.3390/cells9112395

**Published:** 2020-10-31

**Authors:** Martine Daujat-Chavanieu, Sabine Gerbal-Chaloin

**Affiliations:** IRMB, University of Montpellier, INSERM, CHU Montpellier, 34295 Montpellier, France; martine.daujat@inserm.fr

**Keywords:** CAR, PXR, regulation, expression

## Abstract

Pregnane X receptor (PXR, NR1I2) and constitutive androstane receptor (CAR, NR1I3) are members of the nuclear receptor superfamily that mainly act as ligand-activated transcription factors. Their functions have long been associated with the regulation of drug metabolism and disposition, and it is now well established that they are implicated in physiological and pathological conditions. Considerable efforts have been made to understand the regulation of their activity by their cognate ligand; however, additional regulatory mechanisms, among which the regulation of their expression, modulate their pleiotropic effects. This review summarizes the current knowledge on *CAR* and *PXR* expression during development and adult life; tissue distribution; spatial, temporal, and metabolic regulations; as well as in pathological situations, including chronic diseases and cancers. The expression of CAR and PXR is modulated by complex regulatory mechanisms that involve the interplay of transcription factors and also post-transcriptional and epigenetic modifications. Moreover, many environmental stimuli affect CAR and PXR expression through mechanisms that have not been elucidated.

## 1. Introduction

Pregnane X receptor (PXR, NR1I2) and constitutive androstane receptor (CAR, NR1I3) are members of the nuclear receptor superfamily that mostly includes ligand-activated transcription factors. Their activity has long been associated with the regulation of drug metabolism and disposition through drug metabolizing enzyme and transporter (DMET) expression modulation [1,2]. It is now well established that PXR and CAR participate in the regulation of physiological and pathological conditions and are implicated in disease development [3,4]. PXR is involved in drug metabolism, bile acid and cholesterol metabolism, inflammation, and cancer. Its more flexible ligand-binding pocket, compared with that of other nuclear receptors, explains the wide spectrum of structurally diverse ligands [5]. CAR can modulate the fate of glucose, lipids, and bile acids, and it is also involved in cell cycle regulation and chemical carcinogenesis. Unlike PXR, CAR can be activated through ligand-dependent and also ligand-independent mechanisms [6].

Considerable efforts have been made to understand PXR and CAR activity regulation, mainly by their cognate ligand(s); however, additional regulatory mechanisms influence their activity (for instance, through regulation of their expression). This aspect has been poorly investigated; thus, in this review we present the data generated in several species and organs on CAR and PXR expression regulation in physiological and pathological conditions.

## 2. CAR and PXR Expression during Development

Little is known on CAR and PXR expression during embryonic and fetal development. In rat liver, *Pxr* mRNA levels are between 2.4 and 6.2% of the adult levels from embryonic day (E) 17 to E21, and 20% of the adult level at day 1 after birth [7]. This level rises gradually to 80% of the adult values by week 4 after birth. The nuclear expression level and activity of the PXR protein at E20 are about 40% of the adult value, suggesting posttranscriptional regulation [7]. During the postnatal period, there is a gradual increase in PXR protein amount, as observed for *Pxr* mRNA [7]. These findings were confirmed by a systematic analysis of the expression of nuclear receptors and their target genes, including cytochrome P450 enzymes (CYPs) in rat during in utero development, birth, neonatal age, weanling, puberty, adulthood, and aging. Specifically, the mRNA expression of *Car* and *Pxr* reaches a plateau at day 28 (beginning of puberty) [8]. Unlike *Pxr, Car* expression strongly decreases at day 800 of life. It has been shown that miR-137 inhibits *Car* expression in mouse neonatal liver. In newborn and adult mice, miR-137 and *Car* expression levels are inversely correlated, suggesting a miR-137 role in *Car* expression regulation at birth [9]. 

In calves, the abundance of *CAR* and *PXR* mRNA progressively increases from birth to day 5 and day 159, while that of their partner retinoic X receptor (RXRα) remains constant [10]. In the marine flatfish *Solea senegalensis*, *sspxr1* mRNA expression is first detected at 18.5 h post-fertilization and its level increases until day 3 post-hatch (dph) [11]. Then, *ssprx1* expression remains low until the onset of metamorphosis (9 dph), but increases progressively during pre-metamorphosis, reaching the highest levels in larvae after metamorphosis (20 dph) [11]. 

In humans, Funakoshi et al. [12] found that *CAR* and *PXR* mRNA expression levels at 20 and 23 weeks of gestation represent 0.2% and 3.8%, respectively, of those observed in adult liver. This suggests very low expression levels in human liver at mid-gestation. Betts et al. also found similar expression levels and showed that they were strongly correlated with *CYP3A7* and *CYP3A4* mRNA expression [13]. As observed in rodents, in human liver tissue samples (fetal, neonatal, young, middle-aged, and elderly), *PXR* mRNA expression is low at the fetal and neonatal stages, increases up to middle age [14], and then decreases to fetal levels in the elderly [15]. In liver, *CAR* mRNA level is relatively high late in the first trimester and early in the second trimester (to gestational day ≈137), although these levels are approximately 100-fold lower than those measured in adult liver [14].

*PXR* mRNA is also detected in fetal intestine (5 to 12 gestational weeks) at levels comparable to those in fetal liver. Surprisingly, *CAR* expression in fetal intestine is much higher than in liver (×10^8^). *PXR* and *CAR* mRNA are detected in fetal adrenal gland, but their expression is much lower than in intestine and liver [15]. 

Stem cell differentiation to hepatocyte-like cells (HLC) is used to reproduce liver development in vitro, and one of the most studied end-points is detoxication [16]. *CAR* and *PXR* are upregulated during stem cell differentiation, but to levels much lower than those observed in primary hepatocytes [17]. Gene copy number analysis showed that *CAR* mRNA expression during human embryonic stem cell differentiation toward HLC reaches values close to those detected in liver during the first gestational trimester. Conversely, *PXR* mRNA remains almost undetectable [12,14]. 

PXR and CAR do not seem to have essential developmental roles in mammals. Indeed, PXR [18] and CAR [19] knock-out (KO) mice are phenotypically normal, unless challenged with potential toxic compounds. Moreover, the liver expression of CAR and PXR remains low during fetal and neonatal life. They expression reaches a plateau at puberty, suggesting a role of sexual hormones in the regulation of their expression. Kennedy et al. proposed that growth and/or sex hormones that control sexual dimorphism [20,21] may act as biochemical regulators of DMET activity via interaction with CAR and PXR [22]. The effect of growth/sex hormones on CAR and PXR expression has been poorly explored.

## 3. PXR and CAR Tissue Distribution

PXR tissue distribution was first studied by Zhang et al. in the rat in 1999 [23]. They found that rat *Pxr* is expressed in liver; intestine; and to a lower extent in kidney, lung, and stomach. However, they could not detect any expression in spleen, heart, brain, and testis [23]. A tissue-specific mRNA expression profile analysis of human nuclear receptors showed that *PXR* is abundant in liver, ≈10 times less abundant in small intestine and colon, and ≈100 times less abundant in stomach and skeletal muscle [24]. In the mouse, *Pxr* expression varies along the intestine; it is similar to that of liver in jejunum, ileum, cecum, and colon, but lower in duodenum [25]. In the pig, *PXR* mRNA level is high in liver, small intestine, heart, kidney and colon [26]. In fish, *pxr* mRNA expression presents similarities with the distribution observed in mammals, with higher levels in liver and intestine [11,27]. The high expression level of PXR in the enterohepatic system explains its crucial role as a sensor of environmental cues [28].

Data are limited on CAR tissue distribution. In the Protein Atlas database (https://v18.proteinatlas.org/), human *CAR* mRNA is predominantly expressed in liver and to a lesser extent in small intestine, duodenum, and kidney, but also in brain, skin, and lung. Human *CAR* mRNA is also detected in colon and the Caco2 cell line (human colorectal adenocarcinoma), whereas mouse CAR protein is present in colon and ileum [29]. Low *CAR* mRNA expression has been described in rat and human lung, but is absent in rabbit and mouse lung [30,31,32].

In mouse liver, *Pxr* and *Car* are strongly expressed in hepatocytes [33]. Moreover, *Car* expression is significantly higher in tetraploid (4N) than octoploid (8N) hepatocytes [34]. *PXR* mRNA is detected in primary human Kupffer [35] and stellate cells [36]. Conversely, it is absent, like *Car*, in primary female mouse Kupffer and endothelial cells [33]. *PXR* and *CAR* mRNA are also detected in human circulating blood cells, CD4+ and CD8+ T cells, CD19+ B cells, and primary CD14+ monocytes [37,38]. *PXR* is also observed in the THP1 cell line (acute monocytic leukemia) [35], in mouse peritoneal macrophages [39], mesenteric adipose tissue [40], and calvaria osteoblasts [41]. PXR and CAR expression profiles in placenta and brain have been reviewed recently [42,43].

## 4. Splicing Variants

Several splice variants of human CAR have been identified [44,45,46], and they might contribute to CAR functional diversity [47,48,49,50]. Among the major splice variants present in human liver, *CAR2* and *CAR3* are estimated to account for 10% and 40% of the total *CAR* transcripts, respectively [45,51]. Both variants encode proteins that contain short insertions within the ligand-binding domain. Unlike the reference CAR isoform, these variants exhibit low constitutive activity, function as ligand-activated nuclear receptors, and may modulate the activity of the reference CAR isoform. *CAR* mRNA splicing variants are expressed in brain, kidney, liver, testis, intestine, adrenal gland, bone marrow, skin, and fetal liver, and each of them shows a tissue-specific expression pattern [46,52]. In some of the human CAR isoforms, the functional domains, for instance the ligand binding domain or the zinc fingers necessary to dock to the CAR binding element in DNA, are modified. Thus, it is possible that some CAR splice variants have unique functions that remain to be identified. 

A mouse CAR2 variant without the C-terminal portion of the ligand binding/dimerization domain cannot transactivate on its own and does not inhibit transactivation mediated by mouse CAR1 [32]. Five alternatively spliced variants of pig CAR also have been described, each of which generates a truncated protein. The pig CAR splice variant 2 has a dose-dependent dominant negative effect on the activity of the reference CAR isoform [53].

Many PXR gene variants have been described in human liver [46,54,55] and intestine [56] with altered transactivation activity towards target genes. The identification of more than 15 PXR splicing and transcript variants in human liver may contribute to the interindividual variability in DMET expression [54,57]. Recent work has focused on four of these variants. Transcript variant 1 (PXR1) and 2 (PXR2) originate from exon 1a and 1b, respectively, and share exon 2 to exon 9. They respond to agonists by activating target gene expression. Conversely, PXR3, which lacks part of exon 5 that encodes the ligand-binding domain, does not induce target gene expression, but plays a dominant negative effect on PXR1 transcriptional activity (reviewed in [58]). The small PXR (sPXR) variant encodes a dominant negative PXR isoform of 37 kDa that represses the function of full length PXR (51 kDa), likely through competition for cofactors such as steroid receptor coactivator 1. PXR1 and sPXR are downregulated in cancer tissue compared to adjacent normal tissue [59]. The expression profiles of the main PXR variants have not been fully studied. Human PXR variants are expressed in adult and fetal liver, heart, colon, small intestine, stomach, adrenal gland, bone marrow, specific brain regions (thalamus and spinal cord) [55], and in primary and secondary sarcoma cell lines [60]. PXR2 mRNA represents approximatively 6 to 15% of all PXR transcripts in liver, with high interindividual variability (from 1% to 60%) [55,61]. PXR3 mRNA represents 0.32% (0 to 3.84%) of all human PXR transcripts [55]. 

Two mouse PXR isoforms were originally described: PXR1 and PXR2. PXR2 shows reduced ligand activation profile compared with PXR1 [57], decreases the basal transcription of *CYP3A4*, and directly represses PXR1 regulatory effects [62]. Similar to human *PXR*, the pig *PXR* gene has multiple splice variants in the ligand-binding domain [26] that represent about 5.3% of all pig PXR transcripts. None of the pig PXR splice variants is active in a luciferase reporter assay, but two of them significantly increase the transactivation of the reference PXR variant in co-transfection experiments [63].

Additional detailed and comparative mechanistic studies are required to predict the effect of PXR and CAR variant expression in physiological and pathological processes.

## 5. Circadian Clock

Biological rhythms are controlled in part by circadian clocks, i.e., transcriptional mechanisms that synchronize the organism to the daily changes in an anticipatory way. The master clock located in the hypothalamus suprachiasmatic nucleus coordinates all peripheral clocks through neuronal connections and hormonal signals. At the molecular level, circadian clocks comprise a network of genes/transcription factors, including CLOCK and BMAL1, and their target genes period (*PER*), cryptochrome (*CRY*), and the orphan nuclear receptor REV-ERBα that forms several cell-autonomous feedback circuits. REV-ERB and retinoic acid receptor-related orphan receptors (RORs) show opposite circadian expression and transcriptional activity [64]. BMAL1/CLOCK heterodimers activate the transcription of clock-controlled genes, including CRY/PER. Consequently, PER and CRY proteins accumulate, inhibit the transcriptional activity of BMAL1/CLOCK, and block their own transcription. Conversely, REV-ERBα represses BMAL expression, whereas RORs induce it [64,65]. 

CAR and PXR, like many other hepatic transcription factors [66,67], exhibit a peak of mRNA expression around the light/dark transition before the beginning of the active period in animals and in relation to their regular feeding patterns. Therefore, they contribute to the temporal detoxification cycle in liver. However, conflicting findings have been reported, depending on the species and the methodology used to assess whether a gene displays a significant day–night oscillation. For example, according to a cosine-wave pattern algorithm, the nuclear receptors *Car*, *Shp*, and *Rxr* are rhythmically expressed in mouse liver, but not *Pxr* [66]. Other studies reported a clear diurnal variation of mouse *Pxr* expression [68,69]. In rats, *Pxr* displays significant daily oscillations. On the other hand, *Car* mRNA expression tends to be higher in the dark than in the light period [70], whereas it clearly exhibits diurnal difference according to another study [71]. Moreover, the circadian variation of *Car*, *Pxr*, and the related *Cyp* genes is sexually dimorphic, with higher expression reported in female over male mouse liver [21]. Sex hormones and STAT5b mediate differences in growth hormone (GH) secretion patterns between males and females, and together with HNF4α regulate the sexually dimorphic expression of CYPs and other liver-expressed genes [20], possibly including nuclear receptors. 

In mouse liver and small intestine, *Car* but not *Pxr* expression is regulated by the circadian PAR domain basic leucine zipper (PARbZip) transcription factors DBP, HLF, and TEF. Ablation of these three genes results in the loss of the rhythmic expression of *Car* and its target genes and higher sensitivity to xenobiotics. CAR-dependent induction of *Cyp2b10* mRNA expression upon exposure to phenobarbital displays a higher circadian accumulation in epithelial intestinal cells (eightfold) compared with hepatocytes [69]. Importantly, the regulation by circadian rhythms of numerous transcriptional regulators of *Car* expression (see Section 8) may indirectly contribute to the non-transcriptional temporal modulation of *Car* expression. Aryl hydrocarbon receptor (AHR) and its heterodimerization partner aryl hydrocarbon receptor nuclear translocator (ARNT), which regulate CAR levels in mouse and human liver, follow the same rhythmic pattern [72,73]. Similarly, BMAL1 participates in the circadian regulation of *Hnf4α* in mice, mainly through action on its P1 promoter [74]. RORα and RORγ also regulate the circadian transcription of DMET-related genes [75]. PGC-1α is rhythmically expressed and activates the expression of BMAL1 and REV-ERBα through ROR co-activation [76]. The rhythmic repression of glucocorticoid receptor transcriptional activity mediates glucocorticoid signaling that follows a circadian pattern [77].

Chronic circadian misalignment is sufficient to disrupt the liver clock and circadian metabolism and to drive the development of non-alcoholic fatty liver disease (NAFLD), non-alcoholic steatohepatitis (NASH), and hepatocellular carcinoma (HCC) in mice, independently of dietary, exogenous, or genotoxic stress [78]. For example, conditions that simulate long-term jetlag in mice induce sympathetic nervous system dysfunction and peripheral clock suppression, resulting in the downregulation of *Nr1h4* (or *Fxr*, the gene encoding bile acid receptor), disruption of bile acid homeostasis, and upregulation of *Car* and transcription factors that stimulate cell proliferation. The transcriptional activation of *Car* promoter may involve AP1 and CREB factors activated in response to sympathetic nervous system–ADRβ–c-AMP–PKA signaling [78].

## 6. Hepatic Functional Zonation

In the hepatic lobule, hepatocytes are organized in trabeculae and perform different functions depending on their location: periportal (PP) or perivenous (PV). PP hepatocytes are mainly responsible for neoglucogenesis, urea formation, and lipid β-oxidation. PV hepatocytes are primarily involved in glycolysis, glutamine synthesis, lipogenesis, bile acid synthesis, and drug metabolism. These enzyme gradients are conditioned by blood flow, oxygen and nutrient gradients, paracrine signaling, contact with other cell types, and extracellular matrix composition (reviewed in [79]). The APC/Wnt/β-catenin signaling pathway has been identified as the main molecular regulator of liver functional zonation [80,81].

In a global gene expression analysis of PP and PV mouse hepatocytes, following collagenase/digitonin perfusion, Braeuning et al. identified CAR and AHR as PV hepatocyte-enriched proteins [82]. In a mouse model of liver-specific β-catenin KO, *Car* and *Ahr* were drastically downregulated in both sexes, whereas *Pxr* mRNA was slightly reduced only in females [83]. Immuno-histochemistry analysis in rodent and human liver showed that CYP2B and CYP3A, the main CAR and PXR targets, were localized in the PV zone. In liver of *Ctnnb1*^−/−^ mice, *Cyp2b10* expression was strongly induced, while *Cyp3a* was modestly affected [83]. Conversely, their induction by exposure to pregnenolone 16α-carbonitrile was comparable in wild type and *Ctnnb1*^−/−^ animals. This suggests that β-catenin may regulate *Cyp2b10* expression in a direct and indirect manner through CAR expression, but has a modest effect on *Cyp3a* and *Pxr* gene regulation. By using mice in which β-catenin is conditionally activated or ablated, Gougelet et al. demonstrated that the drug and bile metabolism pathways are preferentially targeted by β-catenin, partly through CAR and AHR. Following β-catenin activation, transcription factor 4 (TCF-4) and β-catenin showed strong chromatin occupancy on CAR Wnt-responsive elements (WRE), demonstrating its direct regulation by β-catenin [84]. Moreover, β-catenin–TCF/LEF binding activity was identified on the mouse *Pxr* proximal promoter, but β-catenin activation cannot upregulate the *Pxr* promoter activity, suggesting that β-catenin does not regulate, at least directly, *Pxr* expression [85].

Data are limited on β-catenin-mediated regulation of xenoreceptors in humans. In primary human hepatocytes (PHHs), activation of the β-catenin pathway increases *AHR* mRNA expression, but has no effect on rifampicin-mediated CYP3A4 induction. This suggests that PXR is not a direct target of β-catenin, as observed in rodents. *CAR* expression was not explored in this study [86]. In differentiated HepaRG cells, β-catenin is required for AHR-, CAR-, and PXR-mediated induction of CYP1A, CYP2B6, and CYP3A4, respectively [87]. This effect might be mediated through a synergism between xenoreceptors and β-catenin activity, rather than through the regulation of xenoreceptor expression. 

Mouse liver tumors induced by a single injection of the liver carcinogen N-nitrosodiethylamine (DEN) frequently harbor activating mutations in the *Ha-ras* or *B-raf* proto-oncogenes. When DEN is combined with chronic administration of phenobarbital (a liver tumor promoter), tumors show activating mutations in the *Ctnnb1* proto-oncogene [88]. Schwartz’s group reported that gene expression patterns in mouse liver tumors harboring activating mutations in *Ctnnb1* and *Ha-ras* or *B-raf* correspond to those of PV and PP hepatocytes, respectively [89]. *Car* mRNA expression is reduced in *Ha-ras* and *B-raf* mutated tumors compared with normal liver, whereas its expression is not affected in *Ctnnb1*-mutated tumors [90,91,92]. Despite the reduced *Car* mRNA expression level in *B-raf-* and *Ha-ras*-mutated tumors, *B-raf*-mutated tumors respond with pronounced induction to a CAR activator, as observed in normal tissue, whereas induction is weak in *Ha-ras*-mutated tumors [92]. This difference could be explained by the strong extracellular signal-regulated kinase (ERK) phosphorylation in *Ha-ras*-mutated tumors that may reduce CAR nuclear translocation in response to its activators. These data highlight that besides *Car* expression level, the cell context of activation is very important.

Incubation of mouse primary hepatocytes or 70.4 hepatoma cells with increasing amounts of serum causes a concentration-dependent attenuation of PV markers, whereas the expression of PP markers is increased. Epidermal growth factor (EGF) partly mimics the serum effects in hepatoma cells, and EGF effect can be blocked by ERK inhibitors [93]. By examining the effects of mitogen-activated protein kinase (MAPK) in PHHs, Bachleda et al. observed a strong inhibition of *CAR* mRNA expression in response to sorbitol, anisomycin, or EGF, while *PXR* mRNA was much less affected [94]. Transcriptomic analysis showed that *CAR* is among the most downregulated genes in PHHs incubated with EGF. This downregulation is accompanied by inhibition of CYP2B6 induction mediated by CITCO (a direct CAR activator) and a shift towards *PXR*-mediated gene regulation [95].

The zonation pattern is regulated in a complex and coordinated manner. Regulation of mouse *Car* gene expression is controlled by β-catenin and growth factors, specifically EGF that may participate in CAR PV expression. Nevertheless, due to the lack of good antibodies, CAR protein expression pattern has not been explored in tissue sections. In humans and mice, *PXR* gene expression is not regulated, at least directly, by EGF and β-catenin. Like for CAR, PXR zonal expression pattern needs to be investigated in tissues, but this is limited by the poor antibody quality. Single-cell technology, recently used to describe the expression of bile acid metabolizing enzymes, may be a source of information about xenoreceptor expression pattern [96,97]. 

## 7. Nutritional Status and Microbiome Metabolites 

PXR and CAR are involved in the regulation of energy homeostasis [4]. CAR activation improves insulin sensitivity, inhibits lipogenesis and gluconeogenesis, and increases brown adipose tissue energy expenditure [98]. Unlike CAR beneficial effects, PXR activation quite consistently leads to increased hepatic lipid accumulation and promotes the fatty liver phenotype [4]. In agreement, PXR KO improves high-fat diet (HFD)-induced obesity via induction of FGF15 expression, resulting in suppression of bile acid synthesis and reduction of lipid absorption and liver triglyceride levels [99]. *PXR* gene variants might be associated with disease severity in NAFLD and contribute to progress towards more severe disease stages [100]. However, differences in the mechanisms observed between preclinical models and humans question the physiological relevance of findings in animal models. CAR glucose-lowering effect is consistently found in rodents and humans. Conversely, PXR activation leads to opposite effects on gluconeogenesis in rodents and humans [4]. 

On the other hand, several studies have indicated that PXR/CAR function and target gene regulation could be controlled by the nutritional status and cellular energy state. Specifically, it has been shown that CAR expression can be induced during the feeding–fasting switch. Fasting and caloric restriction increase CAR expression and activity, and then CAR coordinates the adaptive response by slowing down energy expenditure and weight loss [101]. Fasting and glucagon increase cyclic adenosine monophosphate (cAMP) and activate PKA signaling, leading to the induction of peroxisome proliferator-activated receptor coactivator-1α (PGC-1α). PGC-1α interaction with HNF4α directly regulates *CAR* gene expression through an evolutionarily conserved HNF4 response element identified in the mouse and human *CAR* promoters, leading to an increase of ligand-independent CAR activity [102]. Wieneke et al. described an alternative or concomitant mechanism [103]. Fasting typically increases the plasma level of free fatty acids that are natural ligands of peroxisome proliferator-activated receptor alpha (PPARα). PPARα controls the expression of a plethora of genes involved in the lipid metabolic pathway. Consistently, PPARα is widely expressed in tissues with high fatty acid oxidation rate, such as liver [104]. Incubation of rat hepatocytes with the PPARα activator WY14643 and experiments in PPARα-deficient mice showed that a PPARα-dependent increase in *CAR* expression is needed to obtain the full response to starvation [103,105]. A conserved DR1 motif in the *CAR* promoter is necessary and sufficient for this control [103]. 

Zhang et al. showed that PPARα ligands, such as clofibrate, increase *Pxr* transcript level in rat hepatocytes [23]. An in silico analysis of the human *PXR* proximal promoter identified several putative binding sites for liver-enriched transcription factors [106] that harbor a functional PPAR responsive element (PPRE). Fasting-mediated upregulation of PXR expression and function in mice is mediated at least partially by PPARα [107]. While PGC-1α and glucagon increase PXR expression and transactivation function, SIRT1 inhibits PXR coactivation by PGC-1α [107]. Fasting also results in the differential expression of pxr in the Senegalese sole [11].

Feeding rhythm drives the circadian transcriptional regulation by the liver clock. The microbiome is essential to integrate the hepatic clock genes and the regulation of metabolic gene expression for optimal liver function. Many bacterial metabolites in the colon lumen activate PXR and CAR, such as the tryptophan metabolite indole-3-propionic acid (IPA) and the secondary biliary acids deoxycholic acid and lithocholic acid [108]. The gut microbiome affects the activity and expression of PXR and CAR and also the expression of their target genes. *Car* and *Pxr* are upregulated in germ-free mice compared with conventional mice [109,110,111]. IPA can activate PXR that regulates aorta and pulmonary vascular vasodilatory function through endothelial nitric oxide synthase (eNOS) modulation. IPA supplementation in germ-free mice also increases *Pxr* mRNA expression in aortic segments, and as an agonist activates PXR to regulate aorta and pulmonary vascular vasodilatory function [112]. Butyrate induces *PXR* transcription in Caco-2 cells [113]. The intestine microbiota plays a role in the ontogeny and establishment of sexually dimorphic liver metabolism through the regulation of GH secretion and sexual maturation [110,114]. The exact mechanism of action of microbiome metabolites remains elusive.

## 8. Transcriptional Regulation by Transcription Factors

PXR and CAR establish a crosstalk with other nuclear receptors or transcription factors to control various signaling pathways (reviewed in [115]). This crosstalk provides an explanation of how physiological stimuli affect xenoreceptor activities and how xenobiotics/drugs may affect physiological functions through xenoreceptor activation. The involvement of transcription factors in CAR and PXR expression regulation is reviewed in the following section and summarized in Table 1.

### 8.1. Hepatic Nuclear Factor 4α

It is acknowledged that HNF4α represents a central regulator of gene transcription in hepatocytes. Mouse models with specific and conditional deletion of HNF4α in hepatocytes show that this transcription factor is crucial for liver early embryonic development [116] and also for adult liver function [117]. 

In *Hnf4α*^−/−^ mouse fetuses, many genes that are essential for a functional hepatic parenchyma are not expressed, including *Pxr* [118]. In mice with conditional *Hnf4α* deletion, *Cyp3a* basal expression is reduced in fetal and adult life, and inducible expression of *Cyp3a* is reduced in adults [119]. *Pxr* is not expressed in fetal liver of *Hnf4α*^−/−^ mice, while *Car* is not detected in fetal liver of wild-type and mutant animals. No difference has been observed in *Pxr* expression in wild-type and *Hnf4α*^−/−^ adult mice. Conversely, *Car* expression is significantly lower in the liver of *Hnf4α*^−/−^ mice than in wild type animals. These results suggest a dichotomy in HNF4α function in adult and fetal liver [119].

In the human HepG2 cell line, *PXR* promoter activity is significantly increased by co-expression of HNF4α through a DR1 element located on the proximal promoter. Moreover, *PXR* mRNA level positively correlates with that of *HNF4α* in human liver tissue samples [120]. By screening a transcription factor siRNA library in pancreatic cancer cells (AsPC-1) that express high PXR levels, Oladimeji et al. identified N-alpha-acetyltransferase (NAA10) as a factor in the transcriptional machinery that regulates PXR [121]. NAA10 interacts with *PXR* promoter and co-immunoprecipitates with HNF4α, suggesting that NAA10 and HNF4α function in a complex to regulate *PXR* transcription.

*HNF4α* locus is transcriptionally regulated through the use of two distinct promoters, P1 and P2, from which six (α1 to α6) and three isoforms (α7 to α9) are generated though alternative splicing of *HNF4α* pre-mRNA (reviewed in [122]). In murine liver, HNF4α7 is expressed mainly in embryos and is almost absent in adult animals, whereas HNF4α1 is almost exclusively detected in adults [123]. In most studies on HNF4α role in gene regulation, the specific function/expression of the different isoforms was not addressed. Only one study described the specific functions of the HNF4α1 and HNF4α7 isoforms. During human liver development, *CAR* mRNA level increases in parallel, and is correlated with *HNF4α1* level in adults, but not with *HNF4α7*. Moreover, *HNF4α7* mRNA overexpression in HCC samples is accompanied by a marked decrease in *CAR* mRNA level. In CV1 cells, HNF4α1 strongly enhances human *CAR* promoter activity, whereas HNF4α7 is a poor activator and acts as a repressor of HNF4α1-mediated transactivation of the *CAR* promoter [124]. PHH transduction by HNF4α7-expressing lentiviruses decreases *CAR* mRNA level [124], while transduction of liver non-parenchymal epithelial cells [125] with HNF4α1-expressing lentiviruses increases *CAR* mRNA expression (unpublished results).

HNF4α is a master regulator of liver gene expression through a complex mechanism that requires its alternative splicing. The balance between the expression of HNF4α1 and HNF4α7 is probably a key event in the regulated expression of hepatic genes.

### 8.2. Glucocorticoid Receptor

Glucocorticoid receptor (GR) activation by physiological concentrations of glucocorticoids leads to increased expression of CAR and PXR, and also of their partner, RXR. Sub-micromolar concentrations of dexamethasone increase *CAR* [126], *PXR*, and *RXRα* [127] mRNA expression in PHHs. The maximum induction is reached after 6 to 12 h of exposure to 100 nM dexamethasone, and is greatly diminished by the GR antagonist RU486. Ketoconazole and miconazole, two antifungal drugs, also inhibit *CAR* and *PXR* mRNA expression in PHHs through their antagonist activity on GR [128]. Dexamethasone-mediated PXR and CAR induction was confirmed at the protein level [126,129]. A functional distal glucocorticoid response element (GRE) has been identified on *CAR* promoter [130], and putative GREs have been predicted by in silico analysis to be present on *PXR* proximal promoter [106]. Co-transfection of a GR expression plasmid with a reporter plasmid that harbor 2.2 kb of the *PXR* proximal promoter increases promoter activity. Conversely, the promoter activity is reduced when using a 1.5 kb sequence of the *PXR* promoter [106]. However, the presence of a functional GRE on *PXR* promoter has not been demonstrated yet, and an indirect effect of GR cannot be ruled out. Recently, Smutny et al. proposed that dexamethasone downregulates the expression of hsa-miR-18a-5p, resulting in the stabilization of the 3′UTR of *PXR* mRNA and its upregulation [131]. Therefore, GR may regulate *PXR* through a dual mechanism that involves the activation of *PXR* promoter and the stabilization of *PXR* 3′UTR via hsa-miR-18a-5p downregulation.

In rat and mouse primary hepatocytes and in the rat hepatoma H4-II-E-C3 cell line, PXR mRNA and protein expression are regulated by GR [129,132,133,134]. A rat pancreatic acinar-like cell line can be differentiated to HLC by incubation with 10 nM dexamethasone for 14 days [135]. Interestingly, mRNA expression of *Pxr*; *Rxrα*; and, to a lesser extent, *Car* is increased following dexamethasone-induced differentiation, leading to fully functional receptors [135]. In zebrafish larvae and adult liver exposed to dexamethasone, prednisolone, and triamcinolone, *pxr* is among the three most upregulated genes [136]. Conversely, in vitro exposure of rainbow trout primary hepatocytes to 1 µM dexamethasone for 24 h significantly reduces *pxr* mRNA expression [27]. At this concentration, dexamethasone might activate PXR, and this might inhibit PXR expression through a feedback mechanism [131].

GR-mediated PXR regulation is not restricted to liver. *Pxr* mRNA is detected in mouse lung following dexamethasone treatment (intraperitoneal injection) for 4 days, but not in control mice. The mouse PXR ligand pregnenolone 16α-carbonitrile (PCN) cannot trigger consistently the increase of Cyp3a mRNA and protein in the lung of control mice, whereas CYP3A levels are significantly increased in mice treated with dexamethasone and PCN [137]. *Pxr* mRNA expression is also upregulated in response to dexamethasone in rat jejunum, ileum, and colon tissue sections [138]. Finally, PXR protein expression was found to be increased in brain–blood barrier capillaries of rats treated in vivo with dexamethasone [68] and in in vitro-stimulated brain endothelial cells [139].

The existence of a GR-(PXR/CAR)-drug metabolizing and transporter signal transmission cascade was proposed due to GR role in *PXR* and *CAR* gene regulation [115]. Consequently, modification of GR expression or activity may strongly affect PXR and CAR expression.

### 8.3. Aryl Hydrocarbon Receptor

AHR is a basic helix-loop-helix/per-Arnt-Sim transcription factor that can be activated by exogenous and endogenous ligands [140]. Besides its numerous cellular functions, the best characterized AHR function to date is the response to xenobiotics through induction of the DMET network (reviewed in [140]). Several pieces of evidence suggest that AHR also regulates CAR and PXR expression through unknown mechanisms.

In mouse liver and extrahepatic tissues, AHR activation increases *Car* mRNA expression with a temporal pattern similar to that of *Cyp1a1*, a known AHR target gene. This induction is further confirmed in PHHs in response to various AHR activators [72]. For instance, benzo[a]pyrene activates *Car* promoter, induces *Car* transcription in rat hepatocytes, and enhances the phenobarbital-dependent induction of *Cyp2b1* [141]. Conversely, *PXR* mRNA expression is reduced after incubation of PHHs with the AHR ligand TCDD for 24 h [142]. This inhibition is reversed by transfection of anti-AHR siRNAs. Moreover, in HepaRG cells and PHHs, AHR activation decreases rifampicin-induced expression of CYP3A4 [142].

### 8.4. Farnesoid X Receptor

Farnesoid X receptor (FXR) is strongly expressed in liver and intestine, and is the master transcriptional regulator of several entero-hepatic metabolic pathways implicated in bile acid, lipid, and glucose homeostasis [143]. Several studies have shown the importance of CAR and PXR in the regulation of bile acid homeostasis [144]. Jung et al. demonstrated that in mice, FXR positively regulates *Pxr* expression [145], leading to upregulation of PXR target genes. Bile acids activate FXR that then blocks the synthesis and promotes breakdown of bile acids through PXR transcriptional activation. The combination of the two mechanisms leads to efficient liver protection against bile acid-induced toxicity. 

### 8.5. Thyroid Receptor

CAR is involved in the metabolism of thyroid hormones (THs), mainly by inducing enzymes involved in their breakdown [101,146]. On the other hand, in a rat model of acute stroke induced by middle cerebral artery occlusion, hypothyroidism after ischemia/reperfusion brain injury was found to be correlated positively with nuclear thyroid receptor (TR) and CAR levels. In vitro experiments indicated that incubation with the TH T3 increases CAR cytoplasmic and nuclear expression [147], as described earlier in rat hepatocyte progenitor cells, called small hepatocytes. The exact mechanism of CAR regulation by TH is unknown and may be indirect because TRα binding to the TRE-like sequence of *CAR* promoter has not been observed [148]. Conversely, PXR does not seem to be affected by THs.

### 8.6. Other Transcription Factors

PXR participates in osteoblast differentiation and represses osteoblast mineralization. Specifically, Kruppel-like factor 4 inhibits osteoblast differentiation while inducing PXR expression in calvaria osteoblasts [41].

Aouabdi et al. demonstrated that estrogen receptor, GR and PPARα have a positive effect on *PXR* expression in experiments in which Huh7 hepatoma cells were co-transfected with the 2.2 kb *PXR* promoter and expression plasmids for ligand-activated transcription factors. Moreover, CAR and PXR significantly decreased the basal expression of the *PXR* promoter construct [106]. However, opposite conclusions were reported by studies in mice. Experiments in *Pxr* and *Car* single KO mice indicated that PXR represses *Car* expression, whereas CAR does not reciprocally inhibit *Pxr* basal expression [149]. Conversely, in the presence of its ligand PCN, PXR self-regulates its expression and upregulates *Car* expression, whereas CAR ligand has no effect [150]. It is unclear whether the differences in the cross-talk between PXR and CAR are due to the different models used or whether they are species-specific.

Analysis of mouse *Pxr* distal promoter (5 kb) indicates that the first 1000 bp are sufficient to confer maximal liver-specific expression. The presence of an in silico predicted E26 transformation-specific sequence 1 (ETS1) site was confirmed by EMSA and ChIP experiments. In transient transfection experiments in Hepa 1-6 cells, ETS1 increases mouse *Pxr* promoter activation by 1.6-fold. Conversely, proteins of the Ikarose family and nuclear factor family (LyF-VI and NF-1) behave as repressors of the mouse *Pxr* promoter activity [85].

Transfection of human *PXR* proximal promoter deletion variants in HepG2 cells allowed for identifying putative activation elements in the −497 and −397 and negative regulatory elements in the −397 to −315 regions [151]. Transcription factors from the Ets family (PU.1 and ETS1), together with PAX5, LEF-1, and c-Jun, lead to the coordinated upregulation of *PXR* gene transcription. The same group identified a composite element (CE) of multiple overlapping *cis*-elements in *PXR* proximal promoter (from −449 to −427) that is involved in the transcriptional regulation of *PXR* basal expression, both in HepG2 and intestinal LS180 cells [152]. DNA–protein interaction studies showed that three binding complexes interact with CE and contain at least Sp1 and heterogeneous nuclear ribonucleoprotein K (hnRNPK) [152].

## 9. Epigenetic and Post-Transcriptional Regulations

Several studies have demonstrated the fact that epigenetic modifications affect *CAR* and *PXR* gene expression through different mechanisms (DNA methylation, histone modifications, and non-coding RNAs). 

### 9.1. Histone Modifications

Active gene transcription is associated with chromatin structure relaxation following histone tail acetylation that allows wider accessibility to DNA-binding proteins. Conversely, histone hypoacetylation is associated with reduced gene transcription. 

Histone 4 (H4) acetylation at the proximal human *CAR* promoter region is promoted by the GR agonist dexamethasone, and inhibited by lipopolysaccharide (LPS) and interleukin-1 β (IL-1β) [153]. This suggests that pro-inflammatory signaling affects the chromatin structure close to the *CAR* promoter and consequently *CAR* transcription.

SWI/SNF is an ATP-dependent chromatin remodeling complex that regulates gene transcription, being is composed of two catalytic ATPases and additional subunits named BRG/BM-associated factors (BAFs). Meng et al. demonstrated that the SWI/SNF complex is recruited via BAF60a to the *Car* promoter in a diet-sensitive manner in mice [154], leading to increased histone H3 acetylation and trimethylation of H3 lysine 4 (H3K4m3) on the *Car* promoter and increased *Car* expression [154]. Mice in which *Baf60a* was ablated are resistant to diet-induced atherosclerosis [154]. Indeed, hepatic *Baf60a* deficiency impairs bile acid metabolism and intestinal cholesterol absorption through downregulation of *Car* and of genes involved in the expression of the alternative bile acid pathway [154]. 

### 9.2. DNA Methylation 

When occurring at a gene promoter, DNA methylation typically represses gene transcription. In human pancreatic cancer cells, CAR was identified among the methylated genes [155]. In HepG2 cells, berberine, an isoquinoline alkaloid, inhibits expression of CAR and of its target genes CYP2B6 and CYP3A4 [156]. Berberine enhances DNA methylation level genome-wide, but reduces it at CpG sites of the CYP2B6 and CYP3A4 gene promoters in HepG2 cells that stably express CAR [156]. CAR promoter methylation was not described [156].

PXR activity is epigenetically regulated by chromatin modifications, DNA methylation, and non-coding RNAs (reviewed in [157]). Little is known about the epigenetic regulation of its expression. In human neuroblastoma cell lines, a CpG island located around exon 3 of *PXR* shows promoter activity, and its methylation status is inversely correlated with *PXR* expression [158]. Habano et al. demonstrated that in colon cancer cell lines, DNA methylation of the CpG-rich sequence of the *PXR* promoter is increased in cell lines with low *PXR* expression [159]. Moreover, lower *PXR* promoter methylation level was observed in colorectal cancer tissues compared with adjacent normal mucosa, suggesting *PXR* mRNA upregulation during carcinogenesis [159]. 

Epigenetic alterations may induce persistent phenotypic changes. For instance, analysis of the DNA methylation status of CpG-enriched sites of the *PXR* promoter in white blood peripheral cells from pregnant women showed hypermethylation during healthy pregnancy and lower methylation in women with intrahepatic cholestasis of pregnancy [160]. This profile was correlated with bile acid profiling, particularly conjugated bile acids [160].

### 9.3. microRNAs (miRNAs)

PXR protein level is not correlated with *PXR* mRNA expression in human liver, indicating the involvement of post-transcriptional regulation [161]. Among the molecular mechanisms of post-transcriptional regulation, miRNAs are important regulators of target genes by binding to complementary regions of transcripts to repress their translation or regulate their degradation. The data available on *CAR* and *PXR* regulation by miRNAs are summarized in Table 2.

miRNA-148a is the first and most studied miRNA implicated in xenoreceptor regulation. It negatively regulates *PXR* gene expression in human liver, intestinal, and oropharyngeal cell lines. Some studies reported a positive correlation of miRNA-148a expression with that of *CAR* and *PXR* in human liver tissues [162], but not others [163,164].

*CAR* and *PXR* mRNA levels are negatively correlated with that of miRNA-34a in human liver tissues, and *PXR* expression is indirectly inhibited by miRNA-34a through inhibition of HNF4α expression [165].

The expression of *PXR* and *CYP3A* is inhibited by miRNA-18a-5p. In human and mouse liver cells, Smutny et al. observed a significant upregulation of miRNA-18a-5p expression following 6h of incubation with the PXR ligands rifampicin and PCN, suggesting a negative feedback regulation in hepatic cells [131]. In contrast, Sharma et al. reported that miRNA-18a-5p is inhibited in LS180 colon adenocarcinoma cells incubated with rifampicin for 6h [166]. These results highlight the tissue-specific regulation and the importance of temporal profiling. 

### 9.4. Long Non-Coding RNAs (lncRNAs)

In HepG2.2.15 cells that stably express hepatitis B virus (HBV), the HBx protein inhibits the expression of the lncRNA F11-AS1 and induces that of *CAR* mRNA [173]. The lncRNA F11-AS1 binds to miR-211-5p, weakening its ability to bind to *CAR* and inhibit its expression [173]. Emerging evidence has demonstrated that lncRNAs can function as competing endogenous RNAs (ceRNAs) for specific miRNAs, regulating their function and downstream targets [175]. For instance, LINC00844 acts as a ceRNA for miR-785-5p and regulates *PXR* and DMET expression in HepaRG cells and PHHs [174].

A lncRNA microarray analysis of human liver samples showed that expression of the lncRNA hepatocyte nuclear factor 1 alpha antisense 1 (HNF1α-AS1), an antisense RNA of HNF1α, was correlated with the mRNA expression of several CYPs and also *PXR* and *CAR* [176]. Alteration of HNF1α-AS1 expression in human Huh7 liver cells by siRNA-mediated knockdown or plasmid overexpression results in significant changes of *PXR* mRNA expression, without any concomitant change of *CAR* and *HNF1α* expression [176]. 

### 9.5. RNA Editing

The adenosine deaminase acting on RNA (ADAR) enzymes catalyze adenosine-to-inosine RNA editing that modulates gene expression and function. Transfection of siRNAs against *ADAR1* but not *ADAR2* in HepG2 cells resulted in a significant increase in *CAR* mRNA and protein expression, as well as in CYP3A4 and CYP2B6 induction [177]. Incubation with the transcriptional inhibitor α-amanitin led to an increase of *CAR* mRNA half-life from 6.6 h to 18.6 h following ADAR1 silencing, demonstrating that ADRA1 inhibition increases *CAR* mRNA stability in a post-transcriptional manner [177]. Moreover, ADAR1 attenuates *CAR* splicing at intron 3 in an RNA editing-independent manner, possibly resulting in *CAR* downregulation.

## 10. Post-Translational Regulation

Masuyama et al. first showed that PXR is degraded by the proteasome system. Indeed, selective inhibitors of the proteasome pathway, including MG-132 and β-lactone, increase the steady state levels of native PXR protein in mouse mammary gland (BALB-MC) cells [178]. PXR degradation depends on its interaction with suppressor for gal 1 (SUG1, a component of the 26S proteasome complex), and is blocked by endocrine-disrupting chemicals. In the presence of cycloheximide, constitutive PXR is rapidly degraded (t1/2, <4 h), whereas the degradation of progesterone-occupied PXR proceeds at a slower rate (t1/2, <8 h) [178]. 

Later, it was shown that RBCK1, an E3 ubiquitin ligase, interacts with human PXR to increase its ubiquitination and to decrease its expression in PHHs and other cell lines [179]. Ong et al., by using mass spectroscopy and a kinome-wide siRNA screen, identified a pathway that regulates human PXR stability via phosphorylation-facilitated ubiquitination by the serine/threonine kinase DYRK2 and the E3 ubiquitin ligase UBR5 [180]. PXR might be a substrate for multiple E3 ligases, as previously described for other proteins.

Human PXR can also be degraded via an increase of calpain activity, adding a new level of complexity to its turnover [181]. Specifically, in LS180 cells pretreated with cycloheximide, PXR half-life was of ≈4 h, close to the value observed in the mouse, and its degradation was inhibited by incubation with a selective calpain inhibitor. However, in contrast to what observed in the mouse, the presence of a PXR agonist (rifampicin) did not significantly affect PXR turnover rate.

Human CAR activity is regulated by the proteasome complex at multiple levels. CAR is ubiquitinated, and proteasomal inhibition leads to intracellular accumulation of ubiquitinated CAR [182]. However, despite its accumulation, CAR transcriptional activity is markedly downregulated [182]. Indeed, in PHHs, proteasomal inhibition enhances the interaction between CAR and HSP90, affecting its nuclear translocation. This demonstrates that the proteasome system plays a critical role in modulating CAR cellular trafficking [182]. Moreover, mammalian two-hybrid screening experiments showed that MG-132 (a proteasome inhibitor) inhibits the interaction between CAR1 and the co-activators SRC1 and GRP1. Finally, as shown for PXR, SUG-1 represses both constitutive and ligand-activated transcriptional activity of CAR1 [182]. The proteasome ability to regulate CAR activity at multiple levels may contribute to fine-tuning CAR-mediated activation of target genes in response to different CAR ligands and indirect activators, such as phenobarbital.

## 11. Pathologies

### 11.1. Inflammation

Inflammation is a protective response to life-threatening insults (e.g., infection and injury). However, long-term dysregulation of inflammatory pathways contributes to many chronic diseases, including cancer, cardiovascular disease, diabetes, obesity, osteoporosis, rheumatoid arthritis, inflammatory bowel disease, asthma, and central nervous system diseases. 

Inflammation and infection affect the activity and expression of DMETs regulated by xenoreceptors. Indeed, during infection or systemic inflammation, liver, due to its strategic position in the body, integrates signals that affect the drug pharmacokinetics and bioavailability. Since 2000, significant advances have been made in unraveling the molecular mechanism that controls the intricacies of inflammation and xenoreceptor-regulated processes. The cross-talk between nuclear receptors and drug metabolism enzymes has been described elsewhere [183], and this review focuses on inflammation-mediated regulation of CAR and PXR expression. 

#### 11.1.1. Liver

Induction of acute phase response by administration of LPS or pro-inflammatory cytokines is characterized by the suppression of DMET expression and activity in liver. This effect is associated with a marked reduction of mouse *Car* and *Pxr* mRNA levels, following intraperitoneal injection of LPS [184]. Administration of PHA40, an inhibitor of nuclear factor κB (NF-κB), with LPS does not significantly alter the endotoxin-mediated downregulation of *Car* mRNA. This suggests that NF-κB does not play a primary role in CAR regulation after endotoxin exposure. Conversely, LPS-mediated inhibition of PXR mRNA and protein expression is significantly attenuated by PHA408. Surprisingly, LPS-mediated *Car* mRNA inhibition is abolished in *Pxr*^−/−^ mice, suggesting a role of PXR in endotoxin-mediated *Car* inhibition [185]. In mouse Kupffer cells, LPS-mediated *Pxr* downregulation is reversed by incubation with selective toxicants [186], antioxidants [186,187,188], and inhibitors of xanthine oxidase and NADPH oxidase [186], suggesting that liver resident macrophages, reactive oxygen species, and xanthine and NADPH oxidases may be involved in this process. On the other hand, selective inhibitors of inducible nitric oxide synthase have no effect [185]. *Pxr* and *Car* mRNAs are also downregulated in response to lipoteichoic acid (LTA) from Gram-positive bacteria, a Toll-like receptor 2 (TLR2) ligand, the signaling of which is mediated through Toll-interleukin 1 receptor domain-containing adaptor protein (TIRAP). Nuclear receptor level is reduced in LTA-treated *Tlr2*^+/+^ and *Tirap*^+/+^ mice, but not in *Tlr2*^−/−^ and *Tirap*^−/−^ mice, demonstrating that TLR2 effect on nuclear receptor expression is mediated by TIRAP [189].

IL-1β, IL-6, and TNF-α are considered the major pro-inflammatory cytokines, with their role in the regulation of CAR and PXR expression having been thoroughly investigated. *CAR* and *PXR* mRNA downregulation in PHHs in response to IL-6 exposure was first described by Pascussi et al. in 2000 [190]. Dose-dependent inhibition was also observed in HepG2 cells (mRNA and protein levels) [191] and in HepaRG cells (mRNA level) [192]. A recent study identified differentiated embryonic chondrocyte-expressed gene 1 (DEC1) as a mediator of IL-6-mediated downregulation of *PXR* and *CAR* in PHHs and HepG2 cells [193]. DEC1 expression is induced in response to IL-6, and DEC1 overexpression has a similar effect as IL-6 on *CAR* and *PXR* expression. DEC1 knockdown reverses *CAR* and *PXR* downregulation by IL-6. Moreover, DEC1 interacts with RXRα, and IL-6 enhances this interaction. IL-6 inhibitory effect on *CAR* and *PXR* expression is also observed in mouse liver [194]. Fasting-mediated increase of *Car* and *Pxr* mRNA level in mouse liver is significantly reduced by short-term (1 h) exposure to IL-6 [195]. Surprisingly, the mRNA expression of *CAR* and *PXR* is increased in response to high IL-6 concentration in primary porcine hepatocytes [196]. Incubation with IL-1β also significantly reduces *CAR* mRNA expression in PHHs by activation of the p65 subunit of NF-κB that interferes with the distal glucocorticoid response element located on *CAR* promoter [153]. In contrast, PXR mRNA and protein expression are not affected in liver of IL-1β or TNF-α treated mice, while only *Car* mRNA is transiently reduced following exposure to these cytokines [197].

#### 11.1.2. Intestine

In human colon biopsies from patients with Crohn’s disease and ulcerative colitis with active inflammation, the mRNA expression of *CAR* and its target *ABCB1* is reduced compared with samples from healthy controls [29]. *PXR* expression also is reduced in colon colitis, and is unaffected [198] or reduced [199] in patients with Crohn’s disease. PXR expression is not affected in mesenteric adipose tissue from patients with Crohn’s disease compared with controls [40]. A strict inverse correlation is observed between colon epithelial PXR expression and NF-κB and IL-8 level in colon biopsies from patients with Crohn’s disease [200]. Addition of TNFα to CaCo-2 cell monolayers results in reduced *PXR* expression and induction of IL-8, suggesting that TNFα-mediated PXR expression inhibition occurs through the NF-κB pathway [199]. In mice with ulcerative colitis, CAR and PXR protein expression levels in liver are reduced [201,202]. Similarly, CAR protein expression is significantly reduced in colon and ileal crypts of mice with dodecyl sulfate-induced colitis [29]. *Pxr* expression is downregulated in the intestine of mice treated with LPS [203] and in transgenic mice that constitutively express TNFα [199]. LPS also reduces *PXR* expression in mouse placenta, and this effect is reversed by antioxidants [204].

It is now well established that the expression of CAR and PXR is regulated negatively during inflammation. In turn, PXR can negatively regulate inflammatory signaling through its ability to inhibit NF-κB activity. Moreover, interaction of the p65 subunit of NF-κB with the dimerization partner RXRα reduces PXR transactivation activity (reviewed in [113]). 

### 11.2. Chronic Diseases

#### 11.2.1. Diabetes and Obesity

Several studies have investigated the expression of drug metabolizing enzymes (DME) and their related nuclear receptors in animals with type 2 diabetes-like syndrome induced genetically or by HFD (Table 3). Contradictory results have been reported, possibly related to differences in the methodologies and animal models used. The level of nuclear receptors, such as CAR, RXRα, and HNF4α, and also of CYP2B and CYP4A, was found to be increased in liver of genetically diabetic db/db mice compared with control C57BL/6 mice [205], and *Pxr* was found to be upregulated in kidney [206]. However, Lam et al. did not observe *Car* and *Pxr* deregulation in liver in the same model [207]. Moreover, the opposite was observed in Zucker fatty rats [208], suggesting that the altered energy metabolism in these animals is not the only cause of CAR changes, or that the position of the mutations in the leptin receptor gene in these two models is important. In male rats with diet-induced obesity (HFD for 12 weeks), the mRNA expression of *Car* and *Pparγ* was increased in the liver compared with rats in the low fat diet group, without significant differences in *Pxr*, *Rxrα*, and *Pparα* expression level [209]. On the other hand, a significant increase in the expression of *Pxr*, *Fxr*, and *Lxrs* and their target genes, but not *Car*, was observed in female Sprague-Dawley rats fed an HFD diet for 13 weeks that were mildly obese or overweight [210]. 

In adult male mice fed a HFD, *Car* and *Pparα* were significantly upregulated during the development of insulin resistance and diabetes, while *Pxr* and *Hnf4α* levels were increased only in the group on HFD for 4 weeks (versus 8 and 18 weeks) [211], as observed in HFD male rats [212]. In another study, *Car* and its target gene *Cyp2b10* were significantly upregulated at 12 weeks, whereas *Pxr* exhibited a moderate but significant increase at 16, 24, and 32 weeks of HFD [213]. In contrast, reduced *Car* and *Pxr* levels and decreased DME expression were observed in male CD1 mice fed a HFD, probably due to a cross-talk between these nuclear receptors and the associated inflammation [214]. In male Tsumura Suzuki obese diabetes (TSOD) mice (a polygenic model that gradually develops obesity and type 2 diabetes), the mRNA expression of *Pxr* and *Pgc-1α*, but not of *Car*, was increased compared with control mice, resulting in increased CYP3A expression and activity. Autoregulation by PGC-1α-activated PXR was proposed as a mechanism for higher *Pxr* expression [215]. To note, *Car* expression was increased in male and female mice fed a HFD for 11 and 36 weeks post-weaning, respectively [216]. Female mice are resistant to the HFD effects [211], suggesting a sexual dimorphic response that could be related to the well documented sexual dimorphism of DME and nuclear receptor basal expression [20,21]. The intestinal and renal expression of *Pxr* also is affected by obesity and diabetes. PXR mRNA and protein levels were significantly increased in duodenum and jejunum, but not ileum, in a rat model of type 2 diabetic mellitus (HFD and streptozotocin treatment) [217]. Gut microbiota metabolites, such as the secondary bile acid lithocholic acid that activates PXR and is elevated in the presence of type 2 diabetic mellitus, could be involved in this upregulation [217]. In normal mouse kidney, *Pxr* is selectively expressed in proximal tubular cells and the promoter is demethylated. In db/db mice, *Pxr* mRNA level is significantly increased, and in the promoter, DNA methylation is further reduced and activation histone marks are enriched. This indicates that in diabetic kidney, *Pxr* is upregulated through alteration of epigenetic regulations [218]. Information on PXR/CAR expression in patients with diabetes and obesity are scarce. A significant upregulation of *PXR* has been observed in patients with chronic kidney disease, including diabetic kidney disease, but also in patients with acute renal dysfunction without rejection after transplantation, suggesting a possible role of PXR in human renal injury [218]. 

Hyperglycemia and hyperinsulinemia in type 2 diabetes mellitus have been linked to NAFLD that can progress to inflammation, fibrosis/cirrhosis, and HCC [219]. High glucose concentration increases PXR expression and activity in HepG2 cells. The AMPK pathway appears to be important for regulating the glucose-induced PXR activity, but does not affect the glucose-dependent regulation of PXR expression [220]. High glucose concentration appears to modulate LPS and IL-6 effect on PXR expression and subcellular localization in hepatocytes [221]. Conversely, long-term exposure of PHHs to hypoglycemic conditions results in increased expression of PXR, CAR, and their target genes [222], in line with data obtained during fasting in rodents [107]. One study investigated in mouse hepatocytes and human hepatic cells the insulin induced-downregulation of carboxylesterase (CES), a PXR target gene, in order to better understand drug–drug interactions and to guide the rational use of drugs in patients with type 2 diabetes. This effect involves the suppression of PXR mRNA and protein expression by insulin in a PI3K/Akt-dependent manner [223]. Interestingly, CES expression variations are associated with PXR expression and activation. Metformin and imitamib both suppress CES through PXR downregulation, but the underlying mechanisms require additional studies [224,225]. In cultured human retinal pigment epithelium cells, high glucose induces iNOS expression that in turn partly suppresses *PXR* transcription, thus inhibiting the expression and activity of P-glycoprotein. Additional studies are needed to determine the functional implication of this crosstalk in the human outer blood–retinal barrier impairment and diabetic retinopathy progression [226].

#### 11.2.2. Fibrosis and Cirrhosis

Worsening of chronic liver disorders, such as chronic HBV and hepatitis C virus (HCV) infection and alcohol abuse, leads to alcohol liver disease (ALD), NASH [227], and chronic cholestatic disease [228] (primary sclerosis cholangitis—PSC, and primary biliary cirrhosis—PBC). These disorders are characterized by hepatocyte damage, recruitment of inflammatory cells, and activation of collagen-producing cells. Data are summarized in Table 3.

In liver specimens from patients with end-stage liver disease (ALD, HCV, and PSC), the expression of CAR, RXR, and AHR, and to a lesser extent PXR, was decreased in comparison with controls, regardless of the pathology, and thus may be one of the factors associated with the reduced liver metabolic capacity and hepatic failure [229]. In patients with chronic HCV infection, the expression of CAR and PXR is largely correlated with the fibrosis stage and the level of metabolic enzymes [230]. The expression of *CAR* and *PXR* is lowest in liver of patients with stage 3 liver fibrosis [231]. CAR levels are unchanged in steatotic, alcoholic, and diabetic cirrhotic liver specimens, while PXR expression is reduced only in liver of patients with cirrhosis and diabetes [232]. Differential expression of specific nuclear receptors correlated with the histologic severity of specific NAFLD features, particularly fibrosis, in pediatric patients enrolled in the TONIC clinical trial. Specifically, 33% of these nuclear receptors, including *CAR*, *PXR*, *GCNF*, *COUP-TF1*, *NURR1*, *PPARs*, *ERs*, *RAR/RXRs*, and *TRs*, were upregulated in samples with fibrosis compared with samples without [233]. As fibrosis, regardless of the diagnosis of NASH, is the only histologic feature that exhibits long-term prognostic relevance in adult patients with NAFLD [234], therapeutic modulation of the expression of these receptors may have important clinical implications [233].

In animal models with CCL4-induced cirrhosis, *Pxr* mRNA expression is reduced in rat liver [235] and mouse liver and small intestine [236]. In these rats, combined treatment with insulin-like growth factor-I and interferon-α can increase *Pxr* expression by improving liver functions and reducing fibrosis [235]. Ginkgolide-A, a natural PXR ligand, improves *Pxr* expression in liver and intestine of cirrhotic mice [236,237]. *Pxr* mRNA expression is also reduced in rats with thioacetamide-induced liver injury [238]. Stroke-prone spontaneously hypertensive rats (SHRSP5/Dmcr strain) fed a high-fat-cholesterol (HFC) diet develop fibrotic steatohepatitis [239] and show reduced *Car* and *Pxr* mRNA expression levels in liver after 8 weeks of this diet [239]. However, CAR and PXR mRNA and protein levels were strongly decreased in HFC-fed males, while they were only slightly affected in HFC-diet fed females [240].

CAR is upregulated in skin and skin fibroblasts isolated from patients with systemic sclerosis. TGF-β induces CAR mRNA and protein expression in fibroblasts from healthy donors in a SMAD-dependent manner. Moreover, CAR agonists increase the activation of canonical TGF-β signaling and exacerbate the fibrosis phenotype [241].

#### 11.2.3. Cholestasis

Recently, Wunsch et al. analyzed *PXR* expression in two chronic cholestatic conditions: PBC and PSC [242]. They found that PXR expression (mRNA and protein) was strongly increased in both pathologies, but this induction did not correlate with cholestasis biochemical features [242]. A previous study found that the expression of *PXR* and *CAR* was reduced to 40–60% in PBC grade III and IV, but this change was not significant [243]. In patients with obstructive cholestasis, PXR and RXR mRNA and protein, as well as CAR protein levels, were markedly increased in comparison with controls [244,245]. *PXR* but not *CAR* mRNA expression was found to be reduced in children with late-stage (but not early stage) obstructive cholestasis caused by biliary atresia [246]. Moreover, after surgical intervention, the expression levels of CAR and PXR were lower in patients with poor prognosis [246]. The anticholestatic activity of bezafibrate was investigated in patients with PBC who showed incomplete response to ursodeoxycholic acid [247]. This study found that bezafibrate is a dual PPAR/PXR agonist with potent anticholestatic activity [247]. It also significantly increases *PXR* mRNA expression, probably through PPARα activation. Upregulation of PXR expression and activity may contribute to bezafibrate anticholestatic activity [247]. 

In bile duct-ligated rats, the nuclear expression of CAR and PXR is significantly reduced [248]. CAR, but not PXR, protein content is restored to normal levels by treatment with dexamethasone that reduces inflammation and oxidative stress [248]. Conversely, *Pxr* mRNA expression was induced in a mouse model of alpha-naphthylisothiocyanate-induced cholestasis [249] and in cholic acid-fed mice [250]. Gabbia et al. demonstrated that CAR and PXR expression levels are influenced by cholestasis severity following bile duct ligation in rats [251]. Indeed, PXR and CAR mRNA and protein expression levels were significantly increased in rats with mild cholestasis, and significantly reduced in rats with severe cholestasis. This suggests that the protective role of CAR and PXR is lost in the late stages of cholestasis following their downregulation [251]. 

### 11.3. Cancer

PXR expression is detected in ovarian [252], colon [253], esophageal [254], and breast carcinoma [255]; in prostate [256] and endometrial cancer [257]; and in sarcoma primary cells [60]. *PXR* is specifically expressed in colorectal cancer stem cells, where it drives the expression of genes involved in self-renewal and chemoresistance [258]. Most chemoresistance-related enzymes, including CYP3A4, P-glycoproteins, and multidrug resistance proteins, are encoded by PXR target genes, and this may contribute to the acquired resistance to chemotherapy in colon, breast, and prostate cancer [259]. 

However, PXR pleiotropic effects in cancer have not been completely elucidated (reviewed in [28]). Recent studies showed that TGF-β plays a key regulatory role in chronic liver diseases, including resistance to chemotherapy in many cancers. Bhagyaraj et al. demonstrated that in the HCC cell line HepG2, TGF-β signaling enhances *PXR* endogenous expression. TGF-β binding to its receptor triggers the activation of the non-canonical ERK signaling pathway. Activated ERK phosphorylates and activates the ETS1 transcription factor and enhances its binding to the *PXR* promoter [260]. The authors proposed that acquisition of drug resistance in response to TGF-β can be mediated through the TGF-β/ERK/ETS1/PXR signaling cascade, leading to increased expression of efflux transporters [260]. Conversely, expression of PXR mRNA and protein and of its target genes is reduced in DEN-induced hepatic cancer in mice, while CAR is not affected and RXRα is induced [261]. PXR downregulation is associated with upregulation of the inflammatory cytokines IL-6 and TNFα. Moreover, HepG2 cells stably transfected with human or mouse PXR display reduced tumorigenic potential in vitro (cell migration, adhesion, invasion), suggesting that high PXR level may reduce tumorigenic potential in liver [261].

CAR role in cancer is still controversial. Phenobarbital, a CAR activator, promotes liver cancer in rodents; however, epidemiological studies have shown that phenobarbital does not increase the incidence of liver tumors in humans [262]. Like PXR, CAR is expressed in cancer cells and cancer stem cells [28,259,263,264]. *CAR* expression is reduced in CD133^+^ brain tumor stem cells, and its activation by CITCO induces its own expression and inhibits stem cell proliferation [265]. In a mouse model of lung carcinogenesis, macrophages and type I and II pneumocytes express CAR, whereas fibroblasts and endothelial cells do not [266]. Moreover, the number of CAR-positive tumor cells was lower in malignant than benign tumor lesions [266]. *CAR* mRNA expression is downregulated in hepatoblastoma and can be used as a predictor to assign unknown samples to the healthy or hepatoblastoma group with 100% accuracy [267]. 

## 12. Xenobiotics

PXR and CAR are activated by many exogenous compounds, including toxic molecules, to protect the body by facilitating their elimination through DMET regulation. While xenoreceptor activation in response to xenobiotics has been extensively studied, the impact on CAR and PXR expression has been poorly investigated. Data are summarized in Table 4.

### 12.1. Nanoparticles

The rapid expansion of nanomaterial technology has led to a growing interest in nanoscience and nanomedicine. As lungs and gastrointestinal tract are in constant contact with the external environment, much higher quantities of nanomaterials are accumulated in liver than in other organs.

Zinc oxide (ZnO) and copper nanoparticles are frequently used as additives in animal feed, representing promising modalities in biomedical research and for clinical applications. Rats fed a diet supplemented with ZnO nanoparticles displayed liver and kidney injury [268]. Moreover, the concentrations of IL-6, INF-γ, and TNF-α in liver were increased, while *Car* mRNA was reduced in a dose-dependent manner. *Pxr* mRNA was only slightly induced [268]. Similarly, in rats, oral exposure to copper nanoparticles induced significant oxidative stress and inflammation, while the mRNA expression of *Car* and *Pxr* was significantly decreased in liver [269]. This was accompanied by activation of the NF-κB, MAPK, and Stat3 pathways. In rat brain, copper nanoparticles induce severe oxidative stress, decrease the expression of most CYP450 enzymes, and inhibit PXR and CAR protein expression [270].

Zebrafish embryos are protected against environmental pollutants, including quantum dots (QDs), thanks to adenosine triphosphate-binding cassette (ABC) transporters. Expression of the *mrp1* and *mrp2* ABC transporters is increased in response to QDs. Moreover, the stronger upregulation of *pxr* and *nrf2*, compared with *mrp* gene induction, could be used as biomarker of QD toxicity [271].

Graphene oxide (GO) is a biocompatible and attractive nanomaterial for drug delivery. In vitro tests demonstrated that the mRNA expression of *PXR* and of its target *ABCB1* is decreased in intestinal LS180 cells and in PHHs after incubation with GO [272]. PXR protein expression also is downregulated in PHHs, independently of the proteasomal pathway [272].

Many studies have investigated the harmful effects of a wide range of nanomaterials in liver, but the underlying mechanisms are still unclear. The analysis of PXR and CAR expression may help to better understand the activity of these new compounds [273].

### 12.2. Environmental Pollutants and Food Contaminants

Endocrine-disrupting chemicals interact with xenobiotics and drug-metabolizing enzymes, representing a global environmental and human health problem [274]. Besides their effect as “endocrine disruptors” that mimic or antagonize the action of endogenous signaling molecules through nuclear receptors [275,276], these molecules might also affect xenoreceptor expression. In juvenile salmon, nonylphenol at environmentally relevant concentrations induce cyp3a and pxr mRNA expression in liver [277]. In rat primary hippocampal cell cultures, nonylphenol neurotoxic and apoptotic effects are accompanied by increased mRNA and protein levels of RXRα, PXR, and CAR [278]. Glyphosate, the most widely used pesticide worldwide, can significantly increase the mRNA and protein expression of CAR, but not of PXR in piglets fed with a glyphosate level based on the maximum residue limits adopted by the Codex Alimentarius Commission. This is not accompanied by an increase of the expression of CAR and PXR target genes [279]. Exposure of HepG2 cells to *cis*-BF, a pyrethroid pesticide, increases PXR mRNA and protein levels, and activates PXR, contributing to *cis*-BF adverse effects on lipid accumulation [280]. 

Arsenic and heavy metals are environmental contaminants implicated in numerous human and animal pathological conditions. In the small intestine of CYP3A4 humanized transgenic mice, arsenite and its metabolites increased *Pxr* mRNA and CYP3A4 mRNA and protein levels [281]. Conversely, in rat and human hepatocytes, co-incubation with arsenite and rifampicin or phenobarbital (two *CYP3A4* inducers) inhibited both basal and rifampicin- and phenobarbital-mediated *CYP3A* mRNA induction, but had no effect on *PXR* expression, and rather inhibited *RXRα* expression [282]. In embryonic zebrafish fibroblasts, heavy metal ions (Ag^+^ and Pb^+^) dramatically (45-fold) induce *pxr* mRNA expression [283], suggesting a role for pxr in the heavy metal detoxication mechanism. 1-Octyl-3-methylimidazolium, a green substitute to conventional solvents, increases both *Cyp3a* and *Pxr* mRNA expression in mouse mammary carcinoma cell lines [284]. In addition to its natural presence at high concentrations in some areas, uranium has several civilian and military applications that could cause contamination of human populations through chronic ingestion. In rats chronically exposed to depleted uranium, *Cyp3a* and *Pxr* mRNA levels were found to be increased in brain, liver, and kidneys, while *Cyp1a* was not affected. Uranium strongly upregulates *Car* mRNA expression in lung [285].

Aflatoxin B1, one of the most common mycotoxins found in human foods and animal feed, is primarily hepatotoxic. In PHHs, aflatoxin B1 at non-cytotoxic concentrations caused a significant increase in *CYP1As, CYP2B6, CYP3As*, and *CYP2C9* mRNA expression [286]. This was accompanied by *PXR*, *CAR*, and *AHR* upregulation [286]. In the same cell model, patulin, another mycotoxin found in fruit, induced *PXR* and also *CYP2B6*, *CYP3As*, and *CYP2C9* expression, but did not modify *CAR* and *AHR* levels [287]. Conversely, the mycotoxin ochratoxin A led to PXR protein decrease in PHHs via a proteasome-independent mechanism, and accelerated *PXR* mRNA degradation via increased miR-148a transcription [288]. In PXR-overexpressing HepG2 cells, a derivative of ochratoxin A that lacks the methyl group of the lactone-ring and the chlorine atom decreased PXR protein expression by 90%, with low cytotoxicity [289]. The PXR expression plasmid contained only PXR coding sequence without the miR-148a recognition element [288], suggesting marginal changes in *PXR* mRNA expression. The mechanism of ochratoxin A-mediated inhibition of PXR expression needs to be explored. 

### 12.3. Drugs 

High doxorubicin doses damage rat gonadal epithelium, and this effect is associated with a strong upregulation of genes implicated in oxidative stress, including *Pxr* (8.2-fold increase) and *Nrf2* (7.4-fold increase) [290]. Paclitaxel induces *Car* mRNA expression in the mouse lung cancer cell line E9, in the presence or absence of CAR ligand [291]. The antiretroviral drug efavirenz and the antituberculosis drug rifampicin reduce *CAR* mRNA expression in HepaRG cells, but not *PXR* mRNA level [292]. In the small benthic fish *Mugilogobius abei*, the mRNA expression of pxr and *cyp3a* was found to be increased following exposure to diclofenac [293]. 

### 12.4. Food Components

PXR and CAR are also activated by many herbal remedies, and are involved in drug–drug and drug–food interactions. The effect of food components has been mainly explored to determine their role in PXR expression in the context of intestinal inflammation. Liu et al. showed that compounds isolated from Chinese herbal medicines can modify PXR expression in the intestinal cell line LS174 [294]. Artemisinin [25], piperidine [295], tanshinone IIA [296], and isorhamnetin [297] are potential agonists and inducers of PXR mRNA and protein expression. In LS174 cells and in mice with dextran sodium sulfate-induced bowel disease, these compounds contribute to reducing the expression of inflammatory mediators and colon inflammation. Isorhamnetin represses the NF-κB pathway, with this potentially contributing to improvements in experimental colitis directly or through induction of PXR expression [297]. A fraction of tannin polymers isolated from the Chinese medicinal herb *Paeoniae radix rubra* inhibits PXR transcription in rat H4IIE liver cells, most likely by disrupting the activity of the transcription factor GR [298]. A combination of fish oil and indomethacin exerts synergistic anti-inflammatory and lipid-lowering effects in plasma and liver via several mechanisms, partly related to PXR upregulation [299]. The probiotic VSL#3 reverses colitis-induced *Pxr* mRNA inhibition in mouse colon and adipose tissue [40]. In female mice fed a HFD and HCD, the combination of purified n–3 fatty acids (n–3) and SC-560 (SC), a cyclooxygenase-1-specific inhibitor, induced both *Pxr* and *Fxr* and reduced *Fgfr4* expression, leading to upregulation of genes involved in bile synthesis and/or detoxification, and ameliorating nonalcoholic fatty liver disease. PXR expression and activation were also increased in Huh7 hepatoma cells incubated with this combination. FXR could mediate at least partly the effect of n–3 + SC through transactivation of PXR and its target genes [300]. A study to determine the role of the nutritional factor alpha-ketoglutarate (AKG) in PXR-mediated improvement of induced colitis showed that PXR activity might be increased through NF-κB downregulation that in turn de-represses PXR. It is unclear whether AKG acts as a regulator to modulate PXR activity [301]. It is important to strictly discriminate between PXR activation and expression in response to these molecules.

Plant extracts also affect CAR expression. Triptolide [302] and xanthohumol [303] negatively affect CAR mRNA and/or protein expression in rat liver and human hepatic cell lines. Moreover, Z-guggulsterone inhibits CAR expression in human microvessel endothelial cells [304]. Catalpol extracted from *Rehmannia glutinosa* reverses triptolide-mediated CAR inhibition [302]. Caffeine, one of the world’s most consumed substances, causes a slight but significant increase of *Car* expression and potentiates the pleiotropic effects of TCPOBOP (the most potent mouse CAR ligand) in mouse liver, including hepatomegaly and hepatocyte proliferation [305].

## 13. Conclusions

The expression of PXR and CAR is influenced by a wide range of physiological, pathological, and environmental stimuli and is controlled by complex regulatory mechanisms that are not well are described and that involve transcription factors and post-transcriptional and epigenetic modifications. 

The observed expression variations are often small, in contrast to what reported for their target genes. R.K. Tyagi’s group proposed that *PXR* gene expression is tightly regulated in physiological conditions by “an interplay between activators and repressors so as to maintain a low cellular level of PXR protein” [151]. In contrast, high PXR expression level could be associated with aberrant homeostasis, diseases, and exposure to some xenobiotics. As described for PXR, CAR expression is low, and its altered in pathological situations and in response to environmental cues. However, changes in CAR and PXR expression are not linear during disease progression. The modification of their expression is probably associated with the disease severity, as suggested by studies on cholestatic disorders. CAR and PXR may exert a protective role in the early stage of hepatic diseases, but this effect may be lost during disease progression. Their expression should be monitored throughout the disease course. 

It is well established that PXR and CAR bind to different chemical compounds and exhibit species and isoform selectivity [307]. Similarly, some of their physiological functions show species differences [4]. However, virtually no study has investigated whether the regulation of their expression is species-specific. It should be noted that regulatory pathways, such as the GR, NF-kB, and PGC-1α/PPARα signaling cascades, seem to be conserved at least between rodents and humans. 

Most of the studies on CAR and PXR expression focused on their mRNA expression. Due to the lack of efficient and specific antibodies, their protein expression has been minimally explored. Consequently, many aspects of PXR and CAR protein expression regulation are unknown, for instance protein half-life and degradation, translation efficiency, variants, and impact of post-translational modifications on protein stability. 

The PXR and CAR activities are controlled by a crosstalk with transcription factors and other nuclear receptors. Most of them also modulate the expression of PXR and CAR, a level of regulation that is still poorly studied. Because of their important roles in physiological and pathological conditions, understanding the regulatory mechanisms of CAR and PXR transcription is crucial to decipher their many functional roles in health and in the development of immune, metabolic, and malignant diseases.

## Figures and Tables

**Table 1:** Transcription factors that regulate CAR and PXR expression.

Transcription Factor	Species	Tissue	Effect	Mechanism	Reference
**PXR**					
HNF4α	Mouse	Liver	+		[118,119]
	Human	HepG2 cells	+	DR1-proximal promoter	[120]
NAA10	Human	AsPC-1 pancreatic cancer cell line	+	HNF4 co-factor	[121]
GR	Human	PHHs	+		[127]
		Huh7 cells	+	2.2kb promoter	[106]
	Rat	Primary hepatocytes	+		[129,132,133,134]
		Pancreatic acinar cell line	+		
	Zebrafish	Larvae, liver	+		[136]
	Mouse	Lung	+		[137]
	Rat	Jejunum, ileum, colon	+		[138]
	Rat	Blood–brain barrier, brain endothelial cells	+		[68,69]
			+		[139]
AHR	Human	Hepatocytes	-	AHR dependent	[142]
FXR	Mouse	Liver	+	IR1	[145]
KLF4	Mouse	Calvaria osteoblasts	+		[41]
PPARα	Human	Huh7 cells	+	2.2 kb promoter, PPRE in silico	[106]
	Rat	Hepatocytes	+		[23]
PPARα PGC-1α	Mouse	Liver	+		[107]
CAR-PXR	Human	Huh7 cells	−	2.2 kb promoter	[106]
ETS1	Mouse	Hepa1-6 cells	+	1 kb promoter	[85]
LyF-VI–NF1	Mouse	Hepa1-6 cells	−	1 kb promoter	[85]
ETS1, PAX5, LEF1, c-Jun	Human	HepG2 cells	+		[151]
Sp1, hnRNPK	Human	HepG2, LS180 cells	+	Composite element	[152]
**CAR**					
HNF4α1	Human	Hepatocytes	+		[124]
HNF4α7	Human	Hepatocytes	+	Poor activator–repressor of HNF4α1	[124]
HNF4α	Mouse	Mouse and human promoters in CV1	+	Co-activation by PGC-1α	[102]
	Human				
GR	Human	PHH	+	Distal GRE	[126,129,130]
	Rat	Pancreatic acinar cell line	+		[135]
AhR	Mouse	Liver and extrahepatic tissue	+		[72,73]
	Human	PHH	+		[72,73]
	Rat	Hepatocytes	+		[141]
TR	Rat	Hepatocyte progenitors	+	Indirect	[148]
TCF/LEF	Mouse	Liver	+	WRE	[85]
PGC-1α	Mouse	Liver, hepatocytes	+	Indirect through HNF4-RE	[102]
PPARα	Rat	Hepatocytes	+	DR1 motif	[103,105]
	Mouse	Liver			
PXR	Mouse	Liver	−/+		[149,150]

PHH: Primary Human Hepatocytes; PPRE: PPAR Responsive Element; WRE: Wnt Responsive Element.

**Table 2:** miRNAs that regulate CAR and PXR expression.

miRNA	Cell Type/Species	Target	Effects	Reference
miRNA-148a	HepG2, LS180 cells	PXR 3′-UTR	Inhibition	[161]
	Human liver	PXR	No correlation in the Chinese Han population	[164]
	Human liver	PXR CAR	No correlation	[163]
	Human liver	PXR CAR	Positive correlation	[162]
	LO2 liver cells	PXR	Inhibition	[167]
	Oropharyngeal cancer cell lines	PXR	Inhibition	[168]
miR-34a	Hela, HepG2 cells Human hepatocytes	PXR	Inhibition through HNF4-α	[165]
	Human liver	PXR CAR	Negative correlation	[162]
	Human liver	CAR	Negative correlation	[163]
miR449-a	Hela, HepG2 cells Human hepatocytes	PXR	Inhibition through HNF4-α	[165]
miR-204	Human liver	CAR	Positive correlation	[163]
miR-21	Human liver	CAR	Negative correlation	[163]
mir-150	Human liver	PXR CAR	Negative correlation	[162]
miR27a	Human liver	PXR	Negative correlation	[162]
miR-561	HepG2	PXR CAR	Induction through DAX-1 inhibition	[169]
miR-137	Mouse primary hepatocytes	PXR	Inhibition	[9]
	Neuroblastoma, HCC, and CRC	CAR	Inhibition	[170]
miRNA-30c-1-3p	293T, HepG2, LS180 cells	PXR 3′-UTR	Inhibition	[171]
miR-140-3p	HepG2 cells MHCC97-H cells	PXR 3′-UTR	Inhibition	[172]
miRNA-18a-5p	LS180 cells	PXR	Inhibition	[166]
	Human hepatocytes Mouse liver HepG2 cells	PXR 3′-UTR	Inhibition	[131]
miR-211-5p	HepG2.2.15 cells	CAR	Inhibition through sequestration by the lncRNA F11-AS1	[173]
miR-486-5p	HepaRG human hepatocytes	PXR	Inhibition through sequestration by the lncRNA LINC00844	[174]

**Table 3:** Effect of different pathologies on the expression of nuclear receptors and cytochrome P450 enzyme (CYP) target genes.

Species	Model	S	Age/ Treatment	Organ	Nuclear Receptors and CYP Target Genes	REF
**Diabetes/Obesity**						
Mouse	db/db	M	>10 w	liver	(+) *Car*, *Rxrα*, *Hnf4α*, *Cyp2b10*, *Cyp2c29*, *Cyp4a10*, CAR, PXR, RXRα, HNF4α α, CYP2B10, CYP2C29, CYP4A10	[205]
	db/db	M	22 w	kidney	(−) *Pxr*	[206]
			8 w		(−) *Pxr*	[218]
	db/db	M	10 w	liver	(+) *Cyp2b10, Cyp4a10, Cyp2c29* (0) *Car, Pxr, Cyp3a11*	[207]
			25 w		(+) *Cyp4a10* (−) *Cyp2c29* (0) *Car, Pxr, Cyp3a11, Cyp2b10*	
	TSOD	M	12 w 28 w	liver	(+) *Cyp3a11* (+) *Pxr*, *Hnf4α*, *Pgc-1α*, *Cyp3a11*, CYP3A, CYP2C (0) *Car*	[215]
Rat	Zucker		7–9 w	liver	(−) *Car*, CAR (0) *Rxra*, *Ppara*, *Fxr*, *Lxr*	[208]
	HFD/streptozotocin		8 w/5 w	intestine	(+) *Pxr*, PXR, *Cyp3a2* (−) *Cyp2c*	[217]
	HFD	M	4 w/4 w	liver	(+) *Pxr*	[212]
	HFD	M	5 w/12 w	liver	(+) *Car*, *Pparγ* (0) PXR, RXRα, PPARα	[209]
	HFD	F	6 w/13 w	liver	(+) *Pxr*, *Fxr*, *Lxrs*, *Cyp3a2*, CYP3A2 (0) CAR	[210]
Mouse	HFD	M	5 w/4 w 5 w/8, 18 w	liver	(+) *Car*, *Pparα*, *Pxr*, *Hnf4α*, *Cyp2b10* (+) *Car*, *Pparα*, *Cyp2b10* (0) *Pxr*, *Hnf4a*	[211]
		F	5 w/4, 18 w 5 w/8 w		(0) *Car*, *Pparα*, *Pxr*, *Hnf4α*, *Cyp2b10* (+) *Hnf4a* (0) *Car*, *Pparα*, *Pxr*, *Cyp2b10*	
	HFD	M F	4 w/11 w 4 w/36 w	liver	(+) CAR (+) CAR	[216]
	HFD	M	6 w/4 w	liver	(+) *Fxr*, *Cyp3a11* (0) *Car*, *Pxr*, *Cyp2b10*	[213]
			6 w/12 w		(+) *Car*, *Pxr*, *Cyp2b10*, *Cyp3a11*	
			6 w/16, 24, 32 w		(+) *Pxr*, *Cyp3a11* (0) *Car*, *Cyp2b10*	
	CD1/HFD	M	6 w/14 w	liver	(−) *Car*, *Pxr*, *Cyp2b10*, *Cyp2a4*, *Cyp3a11* (0) *Rxra*	[214]
Human	Chronic kidney disease, acute renal dysfunction		adult	kidney	(−) PXR	[218]
**Fibrosis/Cirrhosis**						
Human	End-stage liver disease (ALD, HCV, PSC)		adult	liver	(−) *CAR*, *RXR*, *AHR*, *PXR*	[229]
	HCV F3 fibrosis		adult	liver	(−) *CAR*, *PXR*	[231]
	Steatosis, diabetes		adult	liver	(0) *CAR*, *PXR*, *FXR*, *SHP*	[232]
	Alcohol cirrhosis				(0) *CAR*, *PXR*, *SHP* (−) *FXR*	
	Diabetic cirrhosis				(−) *PXR*, *FXR (0) CAR*, *SHP*	
	NAFLD—fibrosis		Pediatric	liver	(+) *CAR*, *PXR*, *GCNF*, *COUP-TF1*, *NURR1*, *PPARs*, *Ers*, *TR*, *RAR/RXR*	[233]
Rat	CCl4-induced cirrhosis	M	3 w/12 w	liver	(−) PXR	[235]
	Thioacetamide-induced cirrhosis		200 g/7 w	liver	(−) *Pxr*, *Cyp3a* (−) PXR, CYP3A	[238]
	SHRSP5/Dmcr HFC	M F	10 w/8 w	liver	(−) *Car*, *Pxr* (−) CAR, PXR (−) *Cyp8b1* (−) *CYP8B1* (+) *Cyp7a1*, *Cyp7b1* (+) CYP7A1, CYP7B1	[239,240]
Mouse	CCl4-induced cirrhosis	M	25 g/12 w	liver intestine	(−) *Pxr, Cyp3a*	[236,237]
**Cholestasis**						
Human	Chronic cholestasis PBC PSC		adult	liver	(+) *PXR* (+) PXR	[242]
	PBC grade III, IV		adult	liver	(0) *PXR*, *CAR*, *FXR*, *RXR*, *SHP*, *HNF1α*, *HNF4α* (−) *CYP7A1* (0) *CYP8B1*, *CYP3A4*	[243]
	Obstructive cholestasis		adult	liver	(+) *PXR*, *RXR*, *VDR*, *HNF4α*, *RARα* (+) PXR, RXR, CAR, VDR, *HNF4α*, *RARα* (+) *CYP7B1* (+) CYP7B1, CYP8B1	[244,245]
	Obstructive cholestasis		children	liver	(0) *PXR* (0) *CAR* (−) *FXR*, *CYP7A1*	[246]
	Early stage					
	Late stage				(−) *PXR* (0) *CAR*, *CYP3A4*	
Rat	Bile duct ligation	M	3 w after surgery	liver	(−) PXR, CAR, *Cyp3a1/2*, CYP3Aa1/2	[248]
	Bile duct ligation Mild cholestasis Severe cholestasis	M	2 w after surgery 4 w after surgery	liver	(+) *Pxr*, *Car*, PXR, CAR, *Cyp3A1/2*, CYP3A1/2 (−) *Pxr, Car*, PXR, CAR, *Cyp3A1/2*, CYP3A1/2	[251]
Mouse	ANIT-induced cholestasis	M		liver	(+) *Pxr*, *Pparα* (−) *Fxr*, *Ahr*, *Shp*, *Cyp7a1*, *Cyp8b1*, CYP8B1	[249]
	CA fed mice	M	10 w/5 d		(+) *Pxr*, *Fxr*, *Cyp3a11*, *Cyp7a1* (−) *Car*	[250]

(+) induction, (−) inhibition, (0) not affected; S: sex; M: male, F: female, w: week, d: day; ALD: alcoholic liver disease; CA: cholic acid; HCV: hepatitis C virus; HFC: high-fat cholesterol; HFD: high-fat diet; PBC: primary biliary cirrhosis; PSC: primary sclerosis.

**Table 4:** Xenobiotics that affect CAR and PXR expression.

Compound	Target	Model	Effect/Mechanism	Reference
Nanoparticles				
ZnO	CAR	Rat liver in vivo	Inhibition through inflammatory signaling	[268]
Nano-copper	CAR PXR	Rat liver in vivo	Inflammatory signaling and oxidative stress	[269]
		Rat brain	Inhibition through oxidative stress	[270]
Quantum dots	PXR	Zebrafish embryos	Induction	[271]
Graphene oxide	PXR	PHH LS174 cells	Proteasome-independent inhibition	[272]
Environmental pollutants				
Nonylphenol	PXR	Juvenile salmon	Induction	[277]
	CAR PXR	Rat hippocampal cells	Induction	[278]
Glyphosate	CAR	Piglet liver	Induction	[279]
*cis*-BF pyrethroid pesticide	PXR	Human HepG2 cells	Induction	[280]
Benzo[a]pyrene	CAR	Rat hepatocytes	Induction	[141]
Arsenic	PXR	Mouse small intestine	Induction	[281]
Arsenite	PXR	Rat and human hepatocytes	No effect	[306]
Heavy metal	PXR	Zebrafish fibroblasts	Induction	[283]
Uranium	PXR	Rat brain, liver, and kidney	Induction	[285]
	CAR	Rat lung	Induction	
1-octyl-3-methylimidazolium	PXR	Mouse mammary carcinoma cell line	Induction	[284]
Food contaminants				
Aflatoxin B1	CAR PXR	PHH	Induction	[286]
Patulin	PXR	PHH	Induction	[287]
Ochratoxin A	PXR	PHH	Proteasome-independent inhibition through miR-148a	[288]
Ochratoxin A derivative	PXR	PXR-overexpressing HepG2 cells	Inhibition	[289]
Drugs				
Diclofenac	PXR	Benthic small fish	Induction	[293]
Doxorubicin	PXR	Rat gonad epithelium	Induction	[290]
Paclitaxel	CAR	Mouse lung cancer cell line E9	Induction	[266]
Efavirenz	CAR	Human HepaRG cells	Inhibition	[292]
Rifampicin				
Food components				
Artemisinin	PXR	Human LS174 cells	Induction	[25]
Piperidine	PXR	Human LS174 cells	Induction	[295]
Tanshione IIA	PXR	Human LS174 cells	Induction	[296]
Isorhamnetin	PXR	Human LS174 cells	Induction	[297]
Probiotics VSL#3	PXR	Colon and adipose/induced colitis mouse model	Reverse colitis-induced PXR inhibition	[40]
A-ketoglutarate	PXR	Induced colitis	Induction	[301]
*Paeoniae radix rubra* extracts	PXR	Rat hepatic cell line H4IIE	Downregulation by disruption of GR signaling	[298]
n−3 fatty acids (n−3) + SC-560	PXR	HFD mice liver Human Huh7 cells	Induction through FXR signaling?	[300]
Fish oil + indomethacin	PXR	Liver	Induction	[299]
Tripolide	CAR	Rat liver Human L-02 and HepG2 cells	Inhibition	[302]
Z-guggulsterone	CAR	Human brain micro-vessel endothelial cells	Inhibition	[304]
Xanthohumol	CAR	Rat liver	Inhibition	[303]
Catalpol	CAR	Rat liver Human L-02 and HepG2 cells	Reverse tripolide-mediated inhibition	[302]
Caffeine	CAR	Mouse liver	Induction	[305]
